# Supplementary material for: Symptoms of Prenatal Depression Associated with Shorter Telomeres in Female Placenta
Source: Int J Mol Sci. 2021 Jul 12;22(14):7458. doi: 10.3390/ijms22147458 (PMC8306199; doi:10.3390/ijms22147458)
Supplement: Supplementary file 1 [file ijms-22-07458-s001.zip › ijms-1264969-supplementary.pdf]

Table S1. Prevalence of obstetric risk factors and association with telomere characteristics. Number (%) is shown. *p* values were assessed with Pearson or Spearman rho correlation. Information for all the stated variables only available for *n*=107/109 participants.

Obstetric risk factor	Prevalence	Association with mean telomere length
History of previous mood disorders	32 (29.4%)	r = 0.04<br>CI = -0.435, 0.87<br>*p* = 0.50
Currently prescribed medication for mood disorder	24 (22%)	r = 0.07<br>CI = -1.007, 1.55<br>*p* = 0.66
Placental abnormalities	6 (5.5%)	r = - 0.02<br>CI = 0.217, 4.52<br>*p* = 0.48
Previous stillbirth	4 (3.7%)	r = - 0.07<br>CI = -2.270, 0.90<br>*p* = 0.39
Previous macrosomic baby	24 (22%)	r = - 0.03<br>CI = -0.799, 0.646<br>*p* = 0.83
Illegal drug use	2 (1.8%)	r = 0.02<br>CI = -1.592, 1.204<br>*p* = 0.78
FGR diagnosis	4 (3.7%)	r = 0.05<br>CI = -1.142, 2.01<br>*p* = 0.58
Proteinuria	14 (12.8%)	r = 0.08<br>CI = -0.559, 1.225<br>*p* = 0.461
Complications during delivery	14 (12.8%)	r = - 0.03<br>CI = -0.287, 0.165<br>*p* = 0.593
Maternal age at booking > 35 years old	29 (26.6%)	r = 0.13<br>CI = -0.214, 0.431<br>*p* = 0.497
Maternal BMI <19.8	3 (3%)	r = 0.06<br>CI = -25.89, 26.13<br>*p* = 0.964
Maternal BMI 19.8-25	27 (24.8%)	r = - 0.19<br>CI = -0.520, 0.201<br>*p* = 0.369
Maternal BMI >25	79 (72.5%)	r = - 0.21<br>CI = -0.139, 0.005<br>*p* = 0.068
GDM	0	N/A
PE	0	N/A
Severe infections	0	N/A

FGR = fetal growth restriction; BMI = body mass index; GDM = Gestational diabetes; PE = Preeclampsia.
